# High-Throughput Sequencing and *De Novo* Assembly of Red and Green Forms of the *Perilla frutescens* var. *crispa* Transcriptome

**DOI:** 10.1371/journal.pone.0129154

**Published:** 2015-06-12

**Authors:** Atsushi Fukushima, Michimi Nakamura, Hideyuki Suzuki, Kazuki Saito, Mami Yamazaki

**Affiliations:** 1 RIKEN Center for Sustainable Resource Science, Yokohama, Kanagawa, 230–0045, Japan; 2 Graduate School of Pharmaceutical Sciences, Chiba University, Chiba-shi, Chiba, 263–8522, Japan; 3 Kazusa DNA Research Institute, Kazusa-Kamatari 2-6-7, Kisarazu, Chiba, 292–0818, Japan; National Key Laboratory of Crop Genetic Improvement, CHINA

## Abstract

*Perilla frutescens* var. *crispa* (Labiatae) has two chemo-varietal forms, i.e. red and green forms of perilla, that differ in the production of anthocyanins. To facilitate molecular biological and biochemical studies in perilla-specialized metabolism we used Illumina RNA-sequencing technology in our comprehensive comparison of the transcriptome map of the leaves of red and green forms of perilla. Sequencing generated over 1.2 billion short reads with an average length of 101 nt. *De novo* transcriptome assembly yielded 47,788 and 47,840 unigenes in the red and green forms of perilla plants, respectively. Comparison of the assembled unigenes and existing perilla cDNA sequences showed highly reliable alignment. All unigenes were annotated with gene ontology (GO) and Enzyme Commission numbers and entered into the Kyoto Encyclopedia of Genes and Genomes. We identified 68 differentially expressed genes (DEGs) in red and green forms of perilla. GO enrichment analysis of the DEGs showed that genes involved in the anthocyanin metabolic process were enriched. Differential expression analysis revealed that the transcript level of anthocyanin biosynthetic unigenes encoding flavonoid 3’-hydroxylase, dihydroflavonol 4-reductase, and anthocyanidin synthase was significantly higher in red perilla, while the transcript level of unigenes encoding limonene synthase was significantly higher in green perilla. Our data serve as a basis for future research on perilla bio-engineering and provide a shortcut for the characterization of new functional genes in *P*. *frutescens*.

## Introduction

Plants can produce a diverse range of secondary metabolites that are beneficial for human health, food, and medicines. When exposed to environmental changes such as drought and water-deficiency, plants can respond to these stresses by producing soluble phenolics, mainly flavonoids (for example, see [1]) and lignins [2]. One group of flavonoids is an anthocyanin pigment [3]. Genes involved in the central flavonoid biosynthetic pathway (see review: [4]), their modification reactions, and their transcriptional regulation have been characterized by the combinatorial approach of transcriptomic and metabolomic profiles with a reverse genetic technique in Arabidopsis [5–8] and other plants [9, 10]. A more detailed genetic, transcriptomic, and metabolomic characterization of pigment plants will lead to a better understanding of the transcriptional regulatory and metabolic systems for anthocyanin biosynthesis.

*Perilla frutescens* var. *crispa* (Labiatae) is a medicinal plant common in Southeast Asia. Among its two chemo-varietal forms, red and green forms of perilla, only red perilla (‘Aka-jiso’ in Japanese) can produce anthocyanins, mainly malonylshisonin [11, 12]. The differential display of mRNA [13] from red and green forms of perilla plants was used for the characterization of genes associated with regulation of the expression of biosynthetic genes [14], for example, the Myb-like gene [15] and the Myc-like gene [16]. Other anthocyanin-related genes have been identified [17–20] and a normalized cDNA library from whole young perilla was constructed and 4,582 uni-expressed sequence tags (uniESTs) were identified [21]. As early methods such as the mRNA differential display, differential hybridization, and serial analysis of gene expression (SAGE) can only monitor a small coverage of the transcript profile, the establishment of fundamental molecular and genetic resources/tools such as DNA microarray- and EST databases remains far from complete in perilla plants.

Recent advances in high-throughput RNA-sequencing technologies (RNA-seq) allow the monitoring of genome-wide transcription, i.e. a complete set of transcripts of an organism (see reviews, [22] and [23]). RNA-seq is applicable to both model organisms with reference genome sequences and to non-model species without an existing reference genome, including crops, trees, and vegetables [24, 25]. It can also detect novel transcribed regions in a genome, small/micro RNAs, and novel alternative splicing patterns. The Medicinal Plant Genomics Resource (MPGR) consortium (http://medicinalplantgenomics.msu.edu/) provides RNA-seq data for 14 medicinal plants including *Catharanthus roseus*; transcriptome data from 23 different tissues in *C*. *roseus* are available [26]. RNA-seq technology is helpful for a better understanding of the perilla-specialized metabolism and its regulation.

Using RNA-seq technology, we analyzed and here described the whole transcriptome map of red and green forms of perilla leaves. We generated over 1.2 billion bases of high-quality short reads using an Illumina sequencer and now demonstrate the suitability of our sequencing for *de novo* transcriptome assembly and the functional annotation of unigenes in perilla leaves. We compared transcript levels in red and green forms of perilla, especially the biosynthetic pathways of anthocyanin and perillyl alcohol. Our findings serve as a basis for future studies on perilla bio-engineering and provide a shortcut to the discovery of new functional genes in *P*. *frutescens*.

## Results and Discussion

### Sample preparation and Illumina sequencing

For the comprehensive characterization of red and green forms of the perilla transcriptome, total RNA samples were isolated from leaves. Using a bioanalyzer we performed DNase treatment and confirmed RNA integrity. Then, the samples were mixed equally. Total RNA was utilized in the mRNA preparation, fragmentation, and cDNA synthesis. After the removal of adaptor sequences and low-quality and ambiguous reads, Illumina sequencing yielded 1,214,546,008 and 1,240,000,000 clean reads from the mRNA pool isolated from *Perilla frutescens* var. *crispa* f. *purpurea* (red perilla) (Table 1) and *P*. *frutescens* var. *crispa* f. *viridis* (green perilla), respectively (S1 Table). The short reads showed mean quality scores 36.2% in red- and 36.3% in green perilla, indicating that our RNA sequencing was adequate for *de novo* assembly.

**Table 1:** Summary of the sequence assembly after Illumina sequencing in red perilla.

	Raw reads	Contigs	Unigenes
Total length (bp)	1,214,546,008	44,923,850	41,869,105
Number of contigs	12,025,208	54,500	47,788
Average length (bp)	101	824	876
Median length (bp)	101	515	580
Max length (bp)	101	12,157	12,157
Min length (bp)	101	201	201
N50 (bp)	101	1,312	1,349

### *De novo* transcriptome assembly of red and green forms of perilla

**Fig 1:**
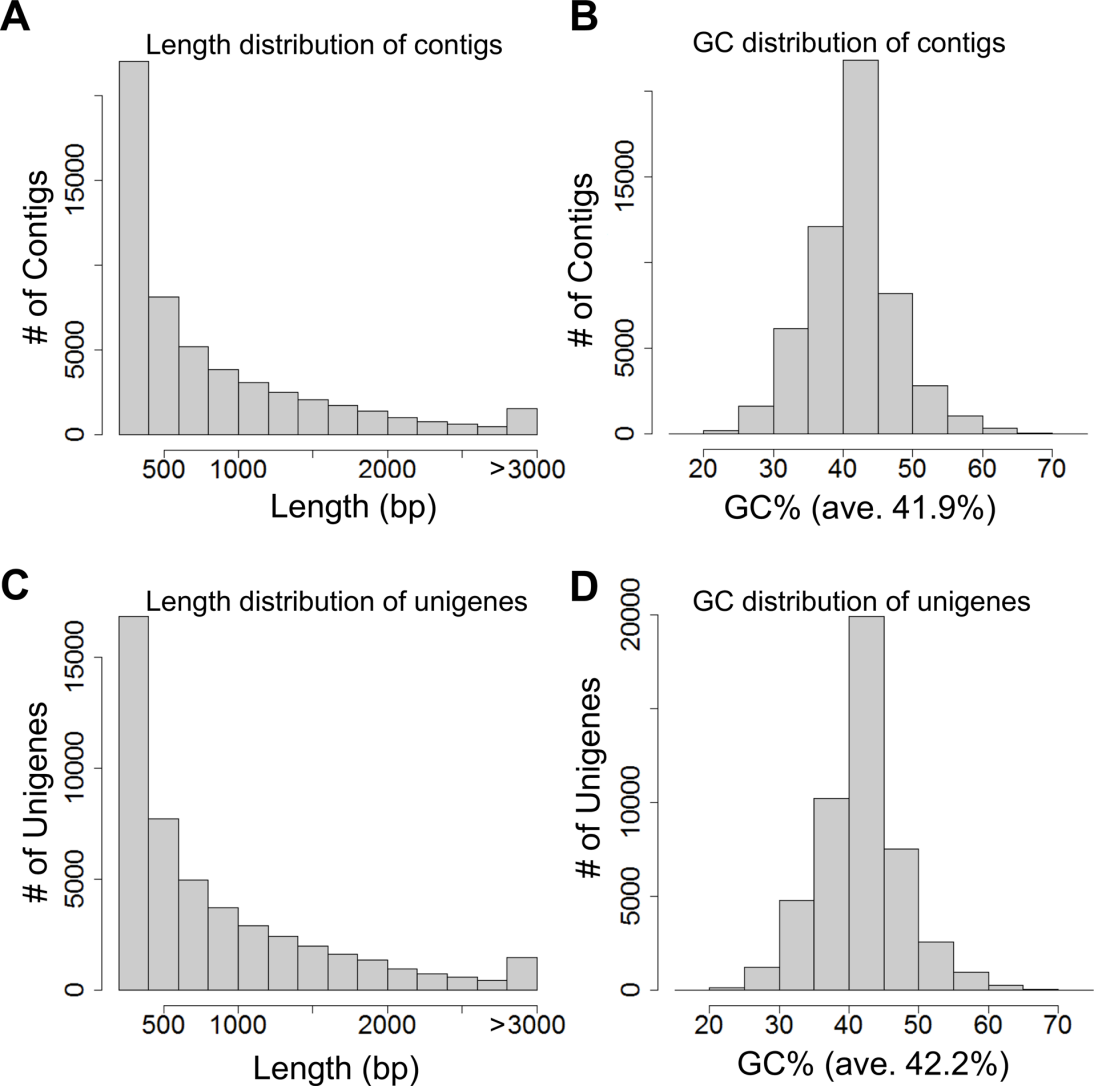
Overview of the *de novo* transcriptome assembly in *Perilla frutescens purpurea* (red perilla). (A and B) Length and GC distribution of contigs assembled from high-quality clean reads by the Trinity program [27]. (C and D) Length and GC distribution of unigenes generated from further contig assembly.

Using the Trinity program [27], all clean reads of red perilla were assembled *de novo* into 54,500 contigs with an average length of 824 base pairs (bp) and an N50 of 1,312 bp (S1 File). In green perilla we obtained 54,445 contigs with an average length of 844 bp and an N50 of 1,368 bp. The length and GC% distribution for all contigs for red and green forms of perilla are shown in Fig 1A and 1B, respectively, and in S1 Fig To estimate expression abundance we used Bowtie [28] and RSEM [29] for the contigs. We obtained 47,788 unigenes with an average length of 876 bp and an N50 of 1,349 bp in red perilla (Table 1) and 47,840 unigenes with an average length of 897 bp and an N50 of 1,399 bp in green perilla (S1 Table). The length and GC content distribution of all assembled unigenes in red and green perilla are shown in Fig 1C and 1D, respectively, and in S1 Fig To provide a general overview of the unigenes we calculated basic statistics. The results showed that in red perilla, 21,174 unigenes were shorter than 500 bp and 1,152 unigenes were longer than 3,000 bp; in green perilla 21,186 unigenes were shorter than 500 bp and 1,257 were longer than 3,000 bp. In 3,909 unigenes of red- and in 3,810 unigenes of green perilla the GC content exceeded 50%.

### Comparison of assembled unigenes and perilla sequences deposited in GenBank

To assess the quality of the assembled unigenes we used all *P*. *frutescens* cDNA sequences available as of December 2014 from NCBI GenBank [30] which contains all 5,911 *P*. *frutescens* sequences (5,538 ESTs and 373 nucleotides). Of the 5,911 cDNA sequences in GenBank, 4,252 (71.9%) could be matched with red perilla unigenes using a cutoff E-value of 10^−10^ (BLASTn). Of 47,788 unigenes, 3,957 (8.3%) matched with 5,911 perilla cDNA sequences, the others were unmatched. There were significant similarities with previously characterized perilla genes encoding glutathione *S*-transferase (AB362191.1: 98.6% identity and E-value = 0.0) [31], anthocyanin 5-*O*-glucosyltransferase (AB013596.1: 97.6% identity and E-value = 0.0) [20], anthocyanin 5-*O*-glucoside-6'''-*O*-malonyltransferase (AF405204.1: 98.9% identity and E-value = 0.0) [32], a WD-repeat-containing putative regulatory protein in anthocyanin biosynthesis (AB059642.1: 98.8% identity and E-value = 0.0) [33], and cytochrome P450 reductase (GQ120439.1: 99.0% identity and E-value = 0.0) [34]. These results indicate that our assembled unigenes have a wide coverage with known perilla cDNA sequences because many unigenes had not been sequenced or had not been assembled correctly.

### Functional annotation and classification of perilla unigenes

**Fig 2:**
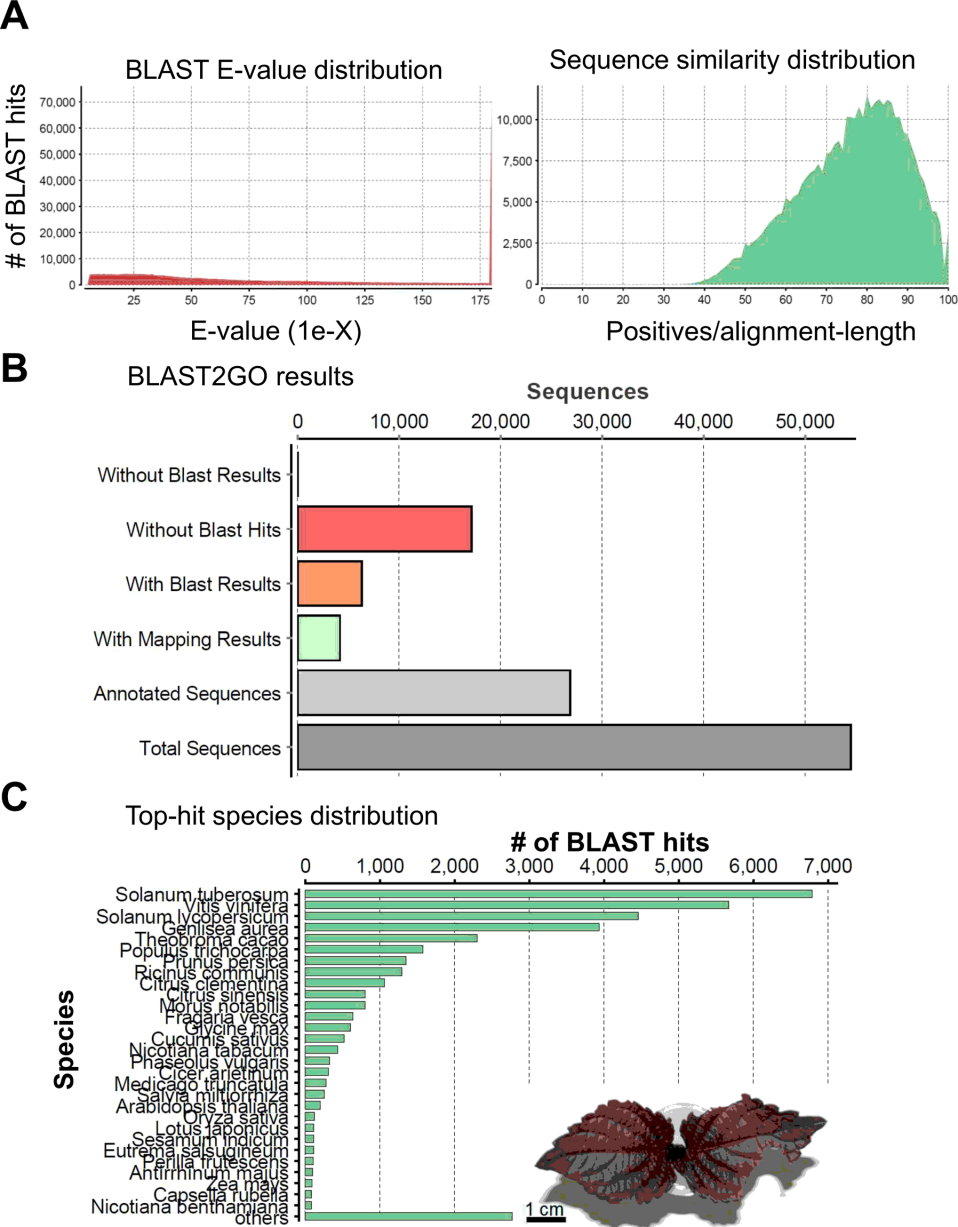
Characterization of the assembled unigenes based on a non-redundant (NR) protein database search in red perilla. (A) (Left panel): E-value distribution of BLAST hits for the assembled unigenes with a cutoff of E-value < 10^−5^. (Right panel): Similarity score distribution of the top BLAST hits for the assembled unigenes with a cutoff of E-value < 10^−5^. (B) Bar chart of the data distribution from BLAST2GO [37]. (C) Species distribution of the top BLAST hits for the assembled unigenes with a cutoff of E-value < 10^−5^.

Next we validated and annotated the assembled unigenes. Our homology search against an NCBI non-redundant (NR) protein database (http://www.ncbi.nlm.nih.gov; formatted on April 7, 2014) was based on the BLASTx program [35] for all unigenes using a cutoff E-value<10^−5^, and the best aligning results were selected to annotate the unigenes. As a result, 81.7% of the aligned sequences in red perilla exhibited significant homology with entries in the NR database (E-value < 1E^-5^) (left panel in Fig 2A). The annotation results for green perilla are shown in S2 Fig Based on the BLAST similarity distribution, 11,630 sequences in red perilla exhibited alignment identities greater than 80% (right panel in Fig 2A). To obtain gene ontology (GO) [36] for the unigenes we used the Blast2GO program v 2.7.1 [37]; it can also assign an Enzyme Commission (EC) number and Kyoto Encyclopedia of Genes and Genomes (KEGG) [38] information based on the BLAST results. The annotation results for red perilla are presented as a bar chart of the data distribution from BLAST2GO (Fig 2B).

A large number of the hits matched the sequences of *Solanum tuberosum* (18.2%), *Vitis vinifera* (15.2%), and *Solanum lycopersicum* (12.0%); other hits were identified within the reference protein databases of *Genlisea aurea* (10.6%), *Theobroma cacao* (6.2%), *Populus trichocarpa* (4.2%), *Prunus persica* (3.6%), *Ricinus communis* (3.5%),*Citrus clementina* (2.8%), and *Citrus sinensis* (2.2%) (Fig 2C). The species distribution of the top BLAST hits for the assembled unigenes from red and green forms of perilla was quite similar (S2 Fig). Although there were many unigenes with no BLAST hits, they may be uncharacterized genes that were not represented in the annotated protein databases or assembled sequences too short to produce hits.

**Fig 3:**
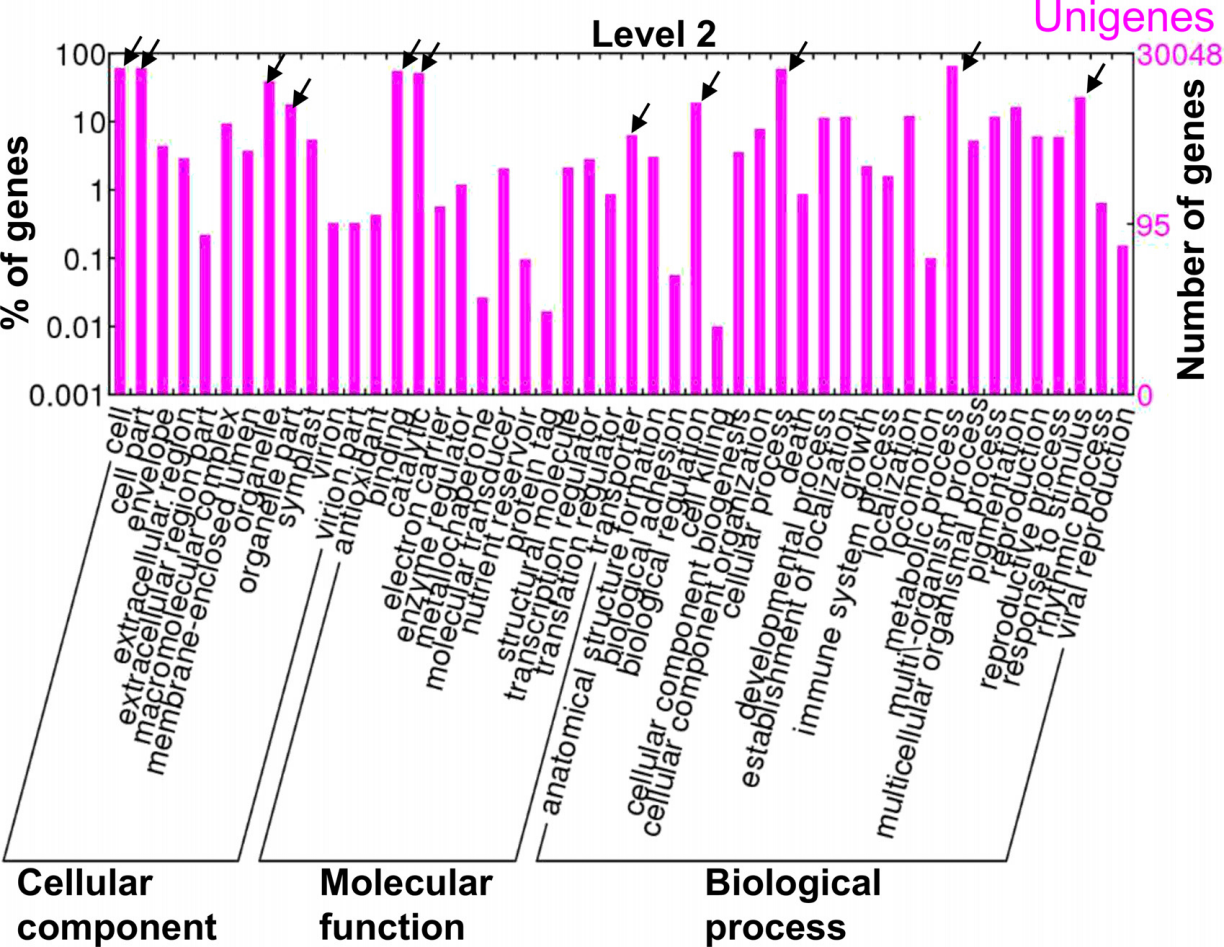
Gene ontology assignments for all assembled unigenes in red perilla. The results are summarized in terms of three functional categories: cellular component, molecular function, and biological process. 30,048 unigenes were categorized by GO terms. The GO terms were visualized using WEGO (http://wego.genomics.org.cn) [39].

We used the BGI WEGO program [39] to perform GO functional classification of all unigenes and of the distribution of gene functions of the species (Fig 3). WEGO can map all of the annotated unigenes to GO terms and identify the number of unigenes involved in each GO term. All 30,048 unigenes in red perilla were categorized by three main GO terms: cellular component, molecular function, and biological process. Within the cellular component, most unigenes were assigned to “cell” and “cell parts”, followed by “organelle” and “organelle part”. Within the molecular function category, the great majority of unigenes was associated with the terms “binding”, “catalytic”, and “transporter”. Within the biological process group, the great majority of unigenes was related to the terms “cellular process”, “metabolic process”, “biological regulation”, and “response to stimulus” (for green perilla, see S3 Fig). All unigenes with functional annotations are presented in S2 and S3 Tables.

### Functional classification by KEGG pathways

**Fig 4:**
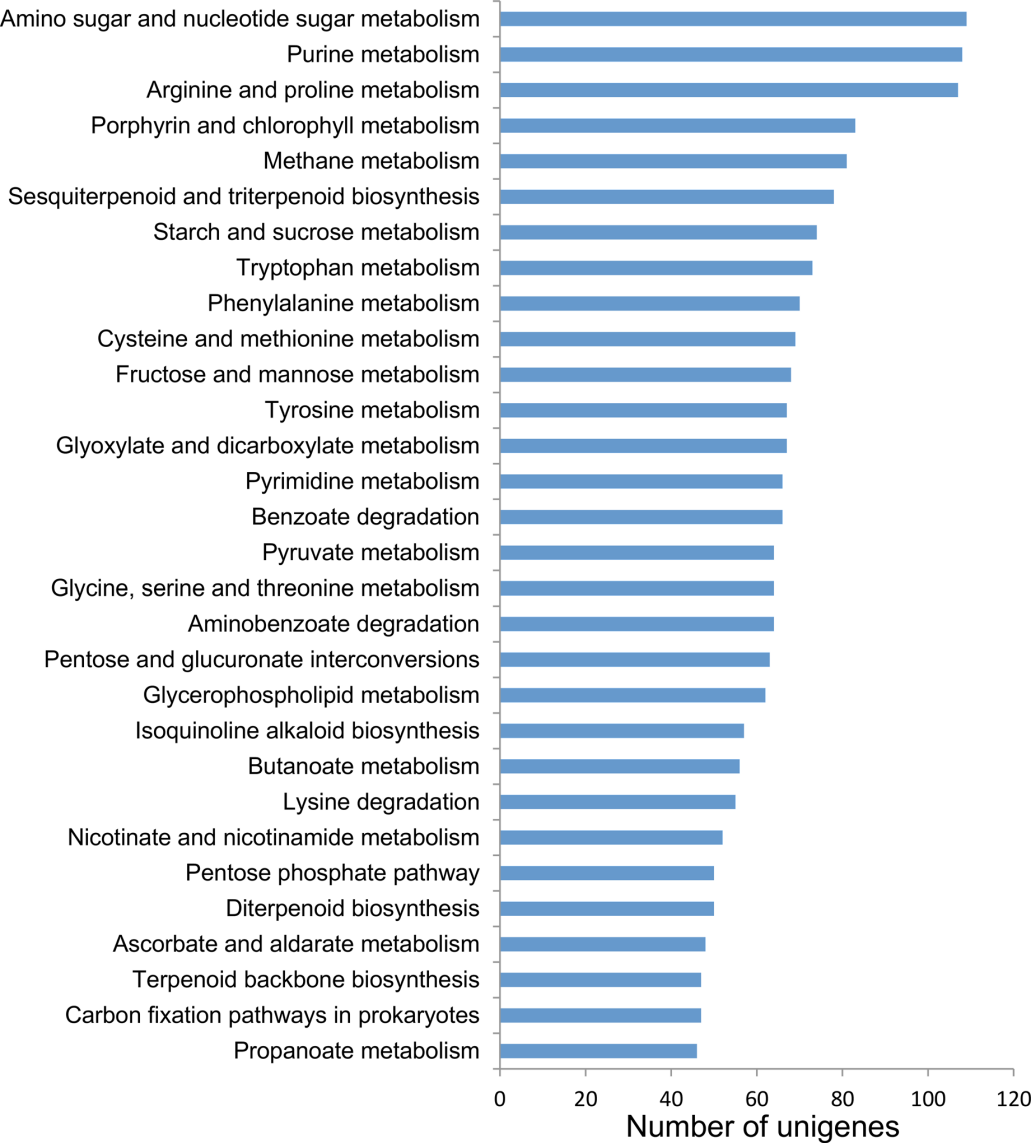
Pathway enrichment analysis of assembled unigenes in red perilla. Annotated unigenes were grouped into 139 KEGG pathways. The top 30 pathways containing unigenes are displayed.

The KEGG pathway database [38] stores the molecular network interactions of cellular components. Pathway-based annotation helps to further understand the biological functions of unigenes. The annotated unigenes in red perilla were grouped into 139 KEGG pathways. Fig 4 represents the top 30 pathways in our enrichment analysis of assembled unigenes in red perilla. The top 3 ranking pathways were amino sugar and nucleotide sugar metabolism (109 unigenes), purine metabolism (108 unigenes), and arginine and proline metabolism (107 unigenes) (Fig 4 and S4 Table). The top 3 ranking pathways were identical for red and green perilla. The number of unigenes associated with the anthocyanin biosynthetic pathway in red and green forms of perilla was 25 and 19, respectively (see also S4 Fig).

### Identification of simple sequence repeats (SSRs)

SSRs, or microsatellites, are ubiquitous repetitive DNA sequences in eukaryotic genomes (see reviews, [40] and [41]). They are important markers for determining functional genetic variation including paternity determination, genetic diversity assessment, population genetics studies, and for the development of a genetic map. To identify SSRs we searched all unigenes in the red and green forms of perilla with MISA [42]. We detected a total of 15,156 SSRs in 12,024 transcripts of red perilla (Table 2 and S5 Table). All SSRs can be classified by the number of repeat units. Di-nucleotide SSRs represented the largest fraction (46.5%) of SSRs identified, followed by mono-nucleotide (36.1%) and tri-nucleotide (17.2%) SSRs. Although only a small fraction of tetra- (120), penta- (12), and hexa-nucleotide (18) SSRs were identified in red perilla transcripts, their number was significant. Our identified SSRs of *Perilla* species may provide potential genetic markers for population genetics and comparative genomics research to enhance our understanding of the genetic control of adaptive traits.

**Table 2:** Statistics of SSRs detected in red perilla.

*Results of SSR searches*	
Total number of sequences examined:	54,500
Total size of examined sequences (bp):	44,923,850
Total number of identified SSRs:	15,156
Number of SSR containing sequences:	12,024
Number of sequences containing more than 1 SSR:	2,460
Number of SSRs present in compound formation:	1,135
*Distribution to different repeat type classes*	
Mono-nucleotide	5,352
Di-nucleotide	7,050
Tri-nucleotide	2,604
Tetra-nucleotide	120
Penta-nucleotide	12
Hexa-nucleotide	18

### Identification of differentially expressed genes (DEGs) in different forms of perilla plants

We identified 68 differentially-expressed genes [false discovery rate (FDR) < 0.05] using the TCC package [43], which is for comparing raw tag count data with a robust normalization method. In S6 Table, we also listed uniquely expressed unigenes in the different forms of perilla plants. The tables feature 22,359 and 22,187 unigenes in the red and green form, respectively. We then performed GO enrichment analysis using a hypergeometric test implemented in BiNGO [44]. This yielded significantly enriched GO functional categories in DEGs compared to the genomic background (S5 Fig). GO functional categories with an FDR < 0.05 were defined as significantly over-represented in DEGs. The top 5 enriched GO terms were “anthocyanin metabolic process (FDR = 1.6E-05)”, “flavonoid metabolic process (FDR = 1.6E-05)”, “phenylpropanoid metabolic process (FDR = 4.6E-05)”, “flavonoid biosynthetic process (FDR = 6.7E-05)”, and “phenylpropanoid biosynthetic process (FDR = 6.7E-05)”. Our identification of DEGs suggests that mainly the biological processes of anthocyanin biosynthesis, but not those of other metabolites, are different in red and green forms of perilla as has been reported previously [12]. The leaves of the red form contain many anthocyanin pigments such as malonylshisonin, shisonin, cis-isomers of malonylshisonin, and peonidin 3-*O*-malonylglucoside-5-*O*-p-coumarylglucoside. Among them, malonylshisonin was the main anthocyanin, representing approximately 70% of the total anthocyanins in red perilla leaves. In contrast, green perilla leaves did not accumulate these anthocyanins [12].

### Exploring gene expressions associated with the biosynthetic pathway of anthocyanins

**Fig 5:**
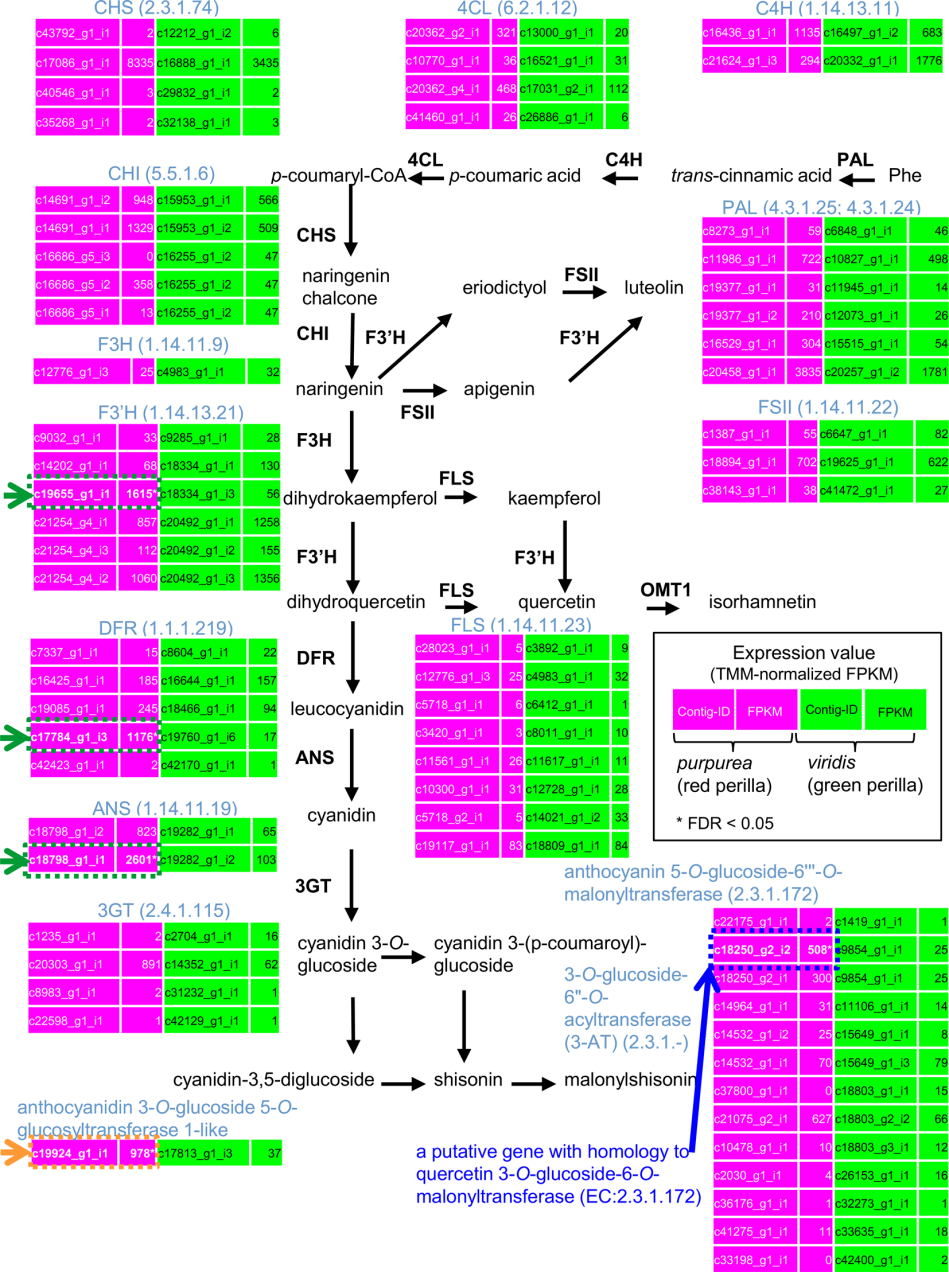
Comparative representation of transcriptome data in red and green forms of perilla plants. Biosynthetic pathways and the expression of unigenes involved in the biosynthesis of phenylpropanoid and flavonoid skeletons are shown. The expression levels (TMM-normalized FPKM values) of unigenes encoding the enzymes of each step are displayed. Homologous genes in red and green perilla represent the reciprocal best-hit BLAST results. Asterisks identify the false discovery rate, (FDR) < 0.05, with the TCC package [43]. TMM, Trimmed Mean of M values [63]; PAL, phenylalanine ammonia-lyase; C4H, cinnamic acid 4-hydroxylase; 4CL, 4-coumaric acid: CoA ligase; ACC, acetyl-CoA carboxylase; CHS, chalcone synthase; CHI, chalcone isomerase; F3H, flavanone 3-hydroxylase; F3’H, flavonoid 3’-hydroxylase; FLS, flavonol synthase; OMT1, O-methyltransferase 1; DFR, dihydroflavonol 4-reductase; ANS, anthocyanidin synthase; 3GT, UDP-glucose: anthocyanidin 3-*O*-glucosyltransferase.

To detect more genes belonging to the relevant biosynthesis pathway of anthocyanin in the transcriptome sequences we carried out a comparative inspection of transcriptome data from red and green forms of perilla plants involved in the biosynthesis of phenylpropanoid and flavonoid skeletons (Fig 5). In this pathway, red and green forms of perilla differed in that red plants manifested higher expression levels of genes encoding flavanone 3’-hydroxylase (F3’H), dihydroflavonol 4-reductase (DFR), and anthocyanidin synthase (ANS) than did green plants (FDR < 0.05) (green arrows in Fig 5). Because F3’H plays a crucial role in pigment biosynthesis in Arabidopsis, mutant plants lacking F3’H (called *tt7*) produce pale brown seeds due to reduced levels of brown pigment [45]. DFR catalyzes the first committed reaction leading to anthocyanin and proanthocyanidin [46]. ANS, also called leucoanthocyanidin dioxygenase (LDOX), can catalyze the formation of cyanidin from leucoanthocyanidin with oxygen and 2-oxo-glutaric acid as co-substrates [18, 47].

In Arabidopsis and maize, a set of transcription factors including MYB [48, 49], basic helix-loop-helix (bHLH) [50], and WD40 [33, 51] plays a central role in the regulation of anthocyanin genes. Tohge et al. [5] suggest that MYB75/PAP1 (PRODUCTION OF ANTHOCYANIN PIGMENT 1) and its homolog MYB90/PAP2 specifically induce the expression of genes associated with the biosynthesis of anthocyanin in Arabidopsis, including DFR and ANS/LDOX. Genes encoding a putative gene with homology to quercetin 3-*O*-glucoside-6-*O*-malonyltransferase (EC:2.3.1.172) (unigene ID: ‘c18250_g2_i2’) (blue arrow in Fig 5) and anthocyanidin 3-*O*-glucoside 5-*O*-glucosyltransferase 1-like (unigene ID: ‘c19924_g1_i1’) (orange arrow in Fig 5) were significantly expressed in red perilla. We also compared the expression levels of previously identified genes including transcription factors and enzymes in red and green forms of perilla (S6 Fig). In red perilla, there was a significant up-regulation (FDR < 0.05) of genes encoding F3G1 (AB103172), bHLH transcription factors associated with the regulation of the flavonoid pathway [52, 53], and glutathione *S*-transferase (AB362191.1) [31].

### Gene expressions involved in the biosynthetic pathway of monoterpenes in red and green forms of perilla

**Fig 6:**
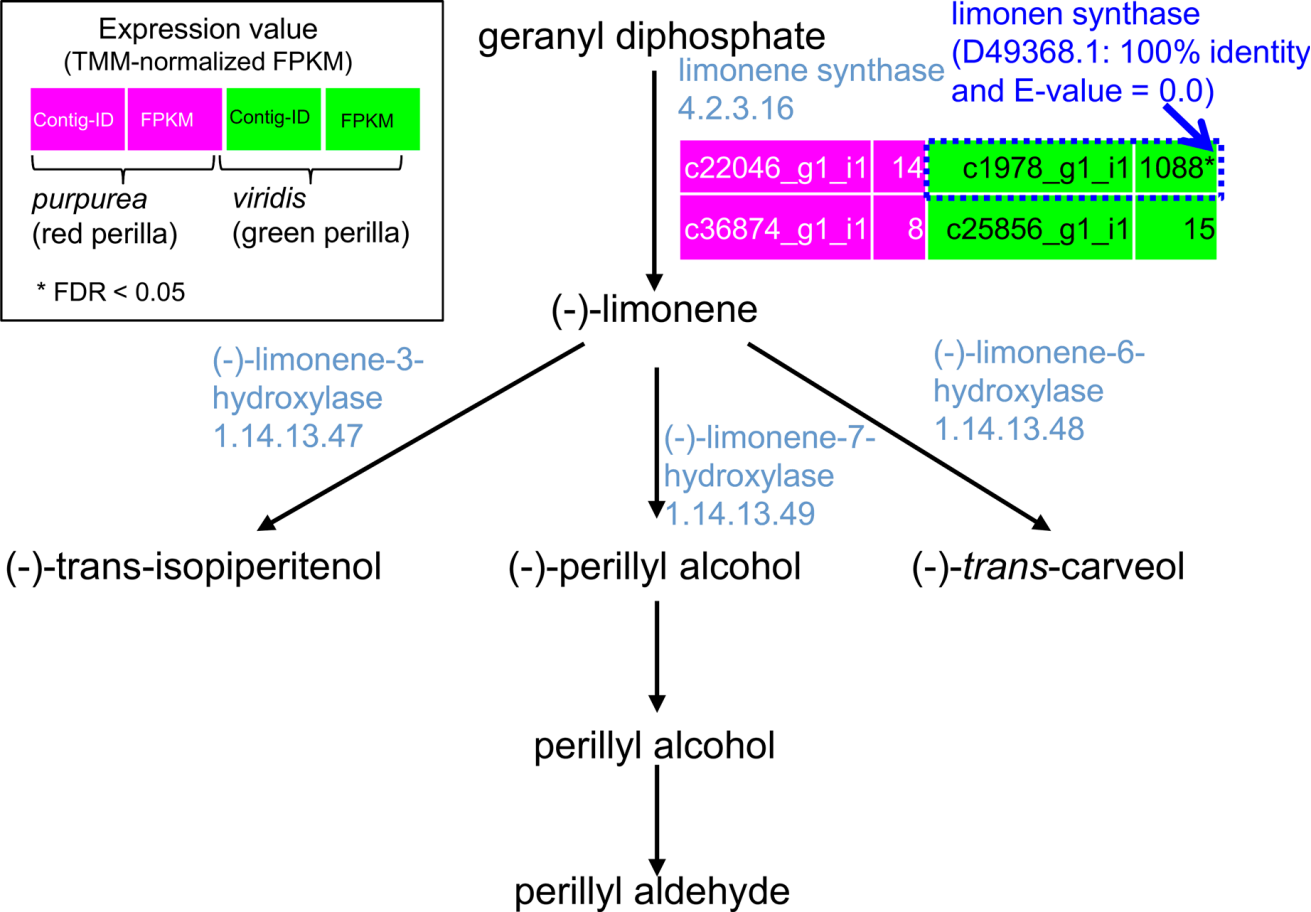
Comparative representation of transcriptome data in red and green perilla plants. Biosynthetic pathways and the expression of unigenes involved in the biosynthesis of perillyl alcohol are displayed. The expression levels (TMM-FPKM values) of unigenes encoding the enzymes of each step are shown. Homologous genes in red and green perilla show the reciprocal best-hit BLAST results. Asterisks indicate the false discovery rate, (FDR) < 0.05, obtained with the TCC package [43]. TMM, Trimmed Mean of M values [63].

Monoterpenes produced by plants play crucial roles in their defense against insects and microbes [54, 55]. Perillyl alcohol, a cyclic monoterpene, is secreted by numerous plant species including lavender, mints, and perilla. The flavor of the perilla herb is characterized by perillaldehyde, an index compound for quality control of the perilla herb in the Japanese Pharmacopoeia (JP). Fig 6 is a comparative representation of transcriptome data related to the biosynthetic pathway of monoterpenes in red and green forms of perilla. Unigene encoding limonene synthase (D49368.1: 100% identity and E-value = 0.0) showed significantly greater up-regulation in green than red perilla. It produces limonene from geranyl diphosphate and is an important step in the biosynthesis of perillaldehyde [56]. One of the major essential oil components of green perilla is limonene. A study that applied gas chromatography—flame ionization detection (GC—FID) showed that the chemical composition of limonene from the aerial parts of red and green forms of perilla was 1.1% and 12.6%, respectively [57]. We think that our results at least partially support the chemical composition of the essential oils of red and green forms of perilla.

In another recent work, Tong et al. [58] analyzed the differences between red and green forms of *P*. *frutescens* var. *crispa* to identify candidate genes involved in leaf color. More studies are needed for a better understanding of the complex regulation of the biosynthetic pathway(s) of anthocyanin and perillyl alcohol in perilla plants and its physiological significance. Although the expression patterns at the protein level must be further investigated, our data and those of others [58], are a basis for future studies on perilla bioengineering and may help to develop an approach for the characterization of new functional genes in *Perilla* species.

## Conclusion

Our study represents comprehensive transcriptome resource for perilla plants that feature two varietal forms of anthocyanin accumulation (red and green forms). Our datasets are an integrated genomic resources for molecular cloning and for identifying genes of interest in perilla. Given the incomplete knowledge on the molecular control mechanism(s) of the biosynthetic pathways associated with anthocyanin and monoterpenes, our transcriptome analysis provides useful information regarding the specialized metabolism of perilla plants.

## Materials and Methods

### Plant materials, RNA isolation, and cDNA synthesis

The plants of *Perilla frutescens* var. *crispa* f. *purpurea* (red perilla) and *P*. *frutescens* var. *crispa* f. *viridis* (green perilla), were grown in the experimental gardens of the Center of Medicinal Resources, Graduate School of Medical and Pharmaceutical Sciences, Chiba University, Chiba, Japan. *P*. *frutescens* var. *crispa* is not an endangered or protected species. Fresh leaves were collected from healthy plants in May 2012. The leaves were frozen by liquid nitrogen and subsequently powdered using a Multi Beads Shocker (Yasui Kikai, Japan). Total RNA was extracted from powdered red and green leaves of *P*. *frutescens* var. *crispa* with the RNeasy Mini Kit (Qiagen, USA), cleaned by ethanol precipitation, and processed using an Illumina TruSeq Prep Kit v2 according to the manufacturer’s protocol (Illumina, San Diego, CA, USA). We used unreplicated data for each form of perilla (i.e., one sample per form).

### Illumina sequencing

cDNA libraries were sequenced on an Illumina HiSeq 1000 sequencer (Illumina Inc., San Diego, CA, USA) and 100-bp paired-end (PE) reads were produced. After the removal of adaptor sequences and ambiguous and low-quality reads, Illumina sequencing resulted in 1,214,546,008 and 1,240,000,000 clean reads from the mRNA pool isolated from red perilla and green perilla, respectively. All raw read sequences are available at the DDBJ Sequence Read Archive [59] under accession number DRA003003.

### Data pre-processing, filtering, and *de novo* transcriptome assembly

For transcriptome assembly we filtered the raw reads and removed adapter sequences, non-coding RNA, low-quality reads with ambiguous ‘N’ bases, and raw reads with an average length less than 20 bases. The Trinity program [27] was used for d*e novo* transcriptome assembly, it combines read sequences of a certain overlap length to form longer fragments without ‘N’ gaps (contigs). We then processed these contigs for read alignment and abundance estimation with Bowtie [28] and RSEM [29]. To calculate unigene expression we used the Fragments Per Kilobase of exon per Million mapped fragments (FPKM) method. In the calculation of gene expression it can exclude sequencing discrepancies and the influence of different gene lengths. The number of unigenes was 47,788 in red- and 47,840 in green perilla at a threshold more than FPKM = 1. The length and GC% distribution of all assembled unigenes are shown in Fig 1C and 1D and in S1 Fig To calculate the GC content and basic statistics values used custom Ruby/Bioruby script [60], the R/Bioconductor package “ShortRead” [61], and “Biostrings” [62].

### Functional annotation and classification of unigenes

We performed a homology search against the NCBI NR protein database (http://www.ncbi.nlm.nih.gov, formatted on April 7, 2014) based on the BLASTx program [35] for all unigenes using a cutoff E-value<10^−5^. The best aligning results were selected to annotate the unigenes. For their further annotation we used the Blast2GO program v 2.7.1 [37] to assign GO terms, an EC number, and KEGG [38] information according to the BLAST results. For visualization of the GO functional classification of all unigenes and the distribution of the gene functions in the different species we used the BGI WEGO program [39]. The microsatellite identification tool (MISA) (http://pgrc.ipk-gatersleben.de/misa/) [42] with its default parameters was applied to identify microsatellites in the unigenes.

### Identification of differentially expressed genes (DEGs)

Differential gene expression analysis between red and green forms of perilla was with the TCC package [43]. Briefly, the main algorithm to identify DEGs with the TCC package is based on the combination of TMM normalization [63] and a DEG identification method [e.g., edgeR [64] and DESeq [65]]. In the DEG identification step, we used a negative binomial test implemented in DESeq [65]. BiNGO [44], a tool to calculate the over-representation of DEGs, was used to analyze significantly over-represented GO categories.

### Global BLAST search against currently available *Perilla frutescens* sequences

To identify putative orthologous genes in red and green forms of perilla plants, all 5,911 *P*. *frutescens* var. *crispa* sequences (5,538 ESTs and 373 nucleotides) were downloaded from NCBI GenBank [30] and then submitted to reciprocal best-hit BLASTn searches against unigenes; the cutoff E-value was < 10^−10^.

## Supporting Information

S1 Fig Overview of our *de novo* transcriptome assembly in *Perilla frutescens* var. *crispa* f. *viridis* (green perilla). (A and B) Length and GC distribution of the contigs assembled from high-quality clean reads by the Trinity program [27].
(C and D) Length and GC distribution of the unigenes generated from further contig assembly.
(PPTX)

S2 Fig Characterization of the assembled unigenes based on a non-redundant (NR) protein database search in green perilla. (Left panel) E-value distribution of BLAST hits for the assembled unigenes with a cutoff of E-value < 10^−5^. (Right panel) Similarity score distribution of the top BLAST hits for the assembled unigenes with a cutoff of E-value < 10^−5^.Bar chart of the data distribution from BLAST2GO [37].Species distribution of the top BLAST hits for the assembled unigenes with a cutoff of E-value < 10^−5^.
(PPTX)

S3 Fig Gene ontology (GO) assignments for all assembled unigenes in green perilla. Results summarized in three functional categories: cellular component, molecular function, and biological process. 29,813 unigenes were categorized by GO terms. The GO terms were visualized using WEGO (http://wego.genomics.org.cn) [39].
(PPTX)

S4 Fig Pathway enrichment analysis of assembled unigenes in green perilla. Annotated unigenes were grouped into 137 KEGG pathways. The top 30 pathways containing unigenes are displayed.
(PPTX)

S5 Fig Significantly enriched GO functional categories in 68 differentially expressed genes in red and green forms of perilla. The colored nodes indicate the significantly over-represented GO categories. The pseudo-colored bar represents the significance [false discovery rate (FDR)].
(PPTX)

S6 Fig Comparative representation of transcriptome data in red and green perilla plants. Previously identified genes encoding transcription factor(s) and enzymes, and the expression of unigenes are shown. The expression levels (TMM-normalized FPKM values) are displayed. Homologous genes in red and green perilla indicate the reciprocal best-hit BLAST results. Asterisks represent the false discovery rate (FDR) < 0.05 obtained with the TCC package [43]. TMM, Trimmed Mean of M values [63].
(PPTX)

S1 File Unigene sequences of *Perilla frutescens* var. *crispa* f. *purpurea (red perilla)* in FASTA format. (FASTA)

S2 File Unigene sequences of *Perilla frutescens* var. *crispa* f. *viridis (green perilla)* in FASTA format. (FASTA)

S1 Table Summary of the sequence assembly after Illumina sequencing in green perilla. (XLSX)

S2 Table List of all annotated unigenes in red perilla. (XLSX)

S3 Table List of all annotated unigenes in green perilla. (XLSX)

S4 Table Functional classification of red (A) and green (B) forms of perilla unigenes by KEGG pathways. (XLSX)

S5 Table Statistics of simple sequence repeats detected in green perilla. (XLSX)

S6 Table Differential expression analysis and identification of uniquely expressed genes in different forms of perilla. Detailed lists of differentially expressed genes (DEGs) up- or down-regulated between red and green forms of perilla.Uniquely expressed genes in red perilla.Uniquely expressed genes in green perilla.
(XLSX)
