# Supplementary material for: High-Throughput Sequencing and De Novo Assembly of Red and Green Forms of the Perilla frutescens var. crispa Transcriptome
Source: PLoS One. 2015 Jun 12;10(6):e0129154. doi: 10.1371/journal.pone.0129154 (PMC4466401; doi:10.1371/journal.pone.0129154)
Supplement: S1 Fig — (A and B) Length and GC distribution of the contigs assembled from high-quality clean reads by the Trinity program [27]. (C and D) Length and GC distribution of the unigenes generated from further contig assembly. (PPTX) [file pone.0129154.s001.pptx]

## Slide 1
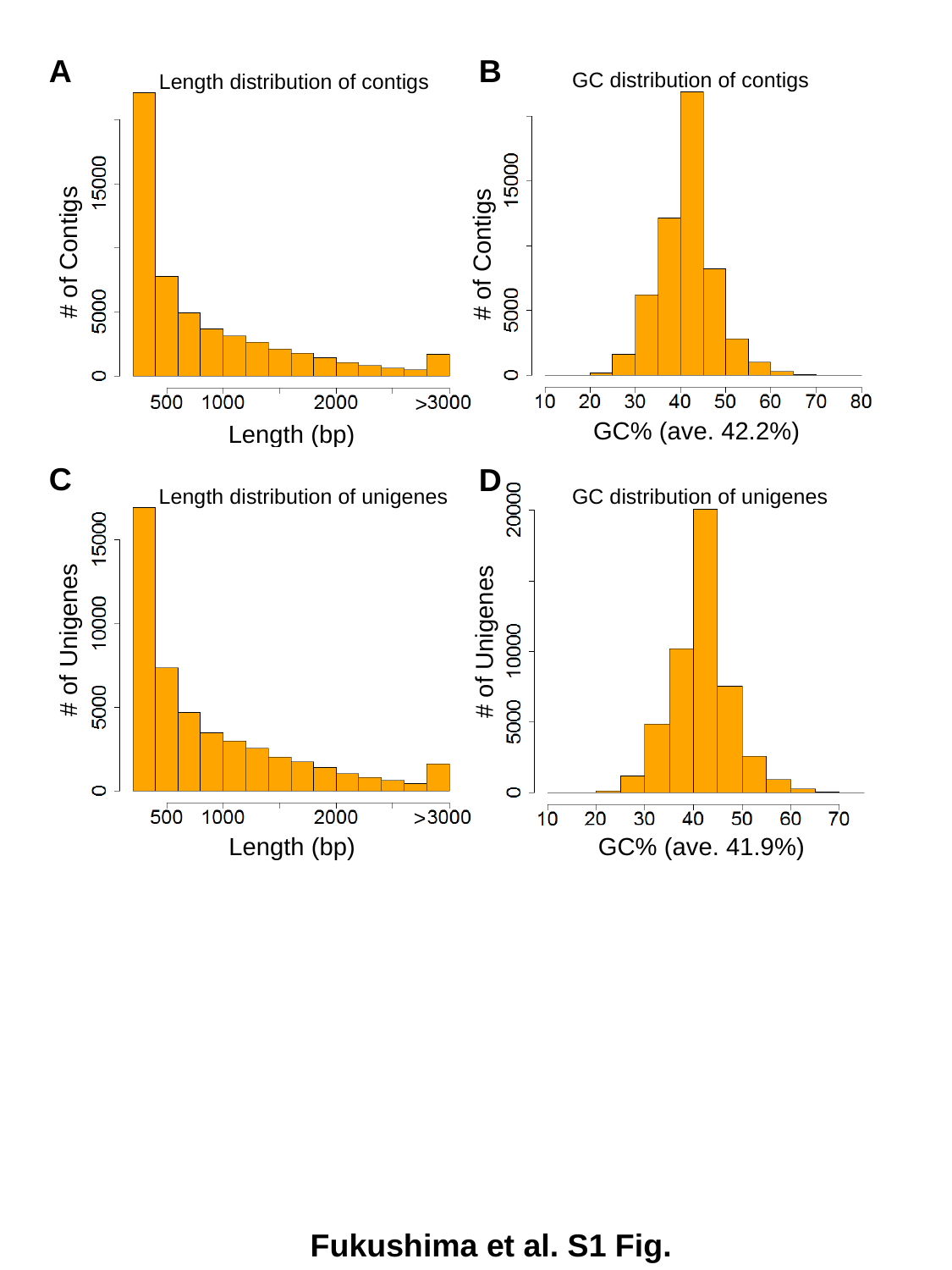

# of Contigs
GC% (ave. 42.2%)
# of Contigs
Length (bp)
A
B
GC distribution of contigs
Length distribution of contigs
>
# of Unigenes
Length (bp)
# of Unigenes
GC% (ave. 41.9%)
C
D
Length distribution of unigenes
GC distribution of unigenes
>
GC% (ave. 43.0%)
Fukushima et al. S1 Fig.
