# Supplementary figures and images for: High-Throughput Sequencing and De Novo Assembly of Red and Green Forms of the Perilla frutescens var. crispa Transcriptome
Source: PLoS One. 2015 Jun 12;10(6):e0129154. doi: 10.1371/journal.pone.0129154 (PMC4466401; doi:10.1371/journal.pone.0129154)

## Slide 1
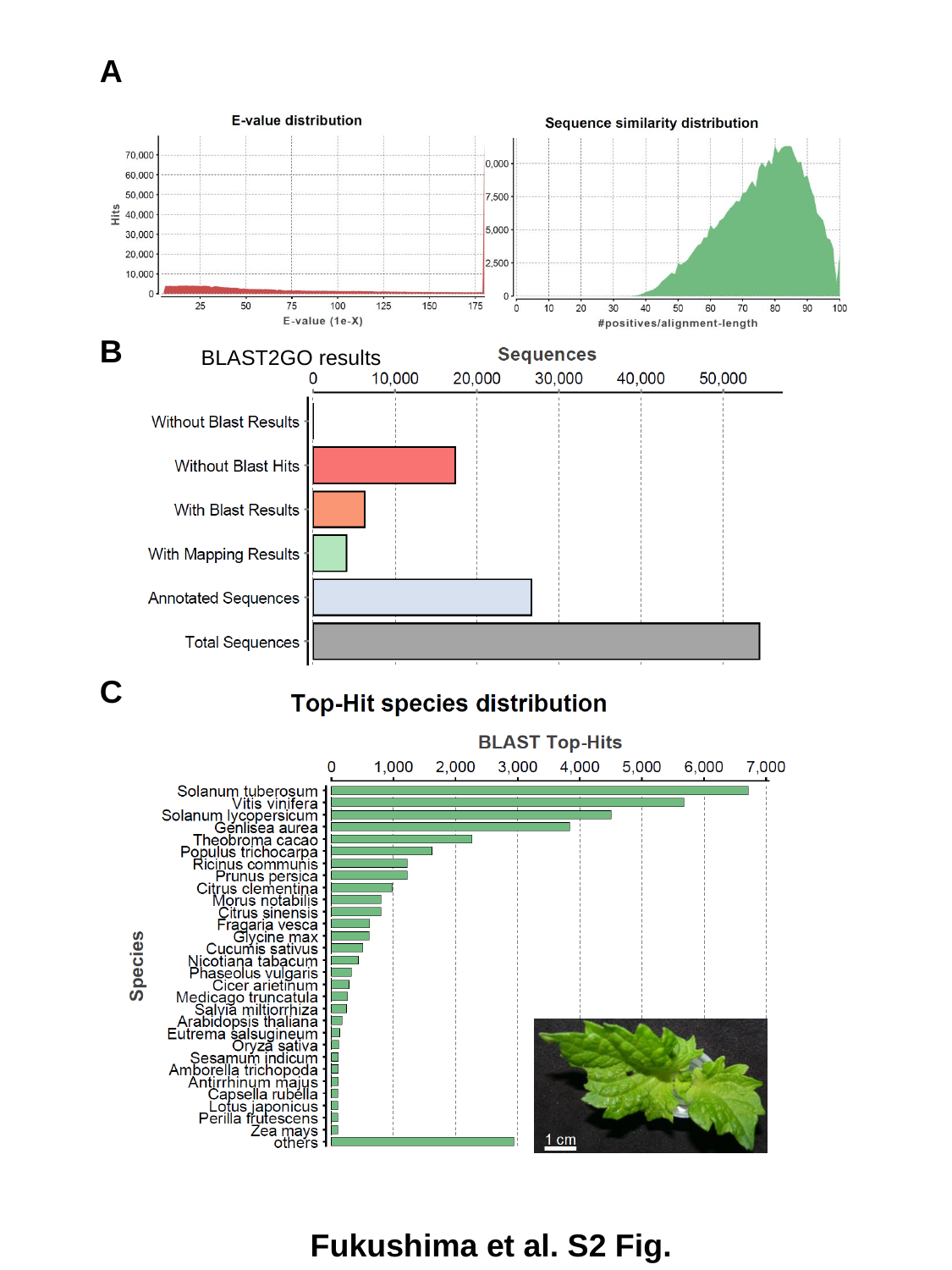

A
B
BLAST2GO results
C
Fukushima et al. S2 Fig.

Supplement: S2 Fig — (Left panel) E-value distribution of BLAST hits for the assembled unigenes with a cutoff of E-value < 10−5. (Right panel) Similarity score distribution of the top BLAST hits for the assembled unigenes with a cutoff of E-value < 10−5.Bar chart of the data distribution from BLAST2GO [37].Species distribution of the top BLAST hits for the assembled unigenes with a cutoff of E-value < 10−5. (PPTX) [file pone.0129154.s002.pptx]

## Slide 1
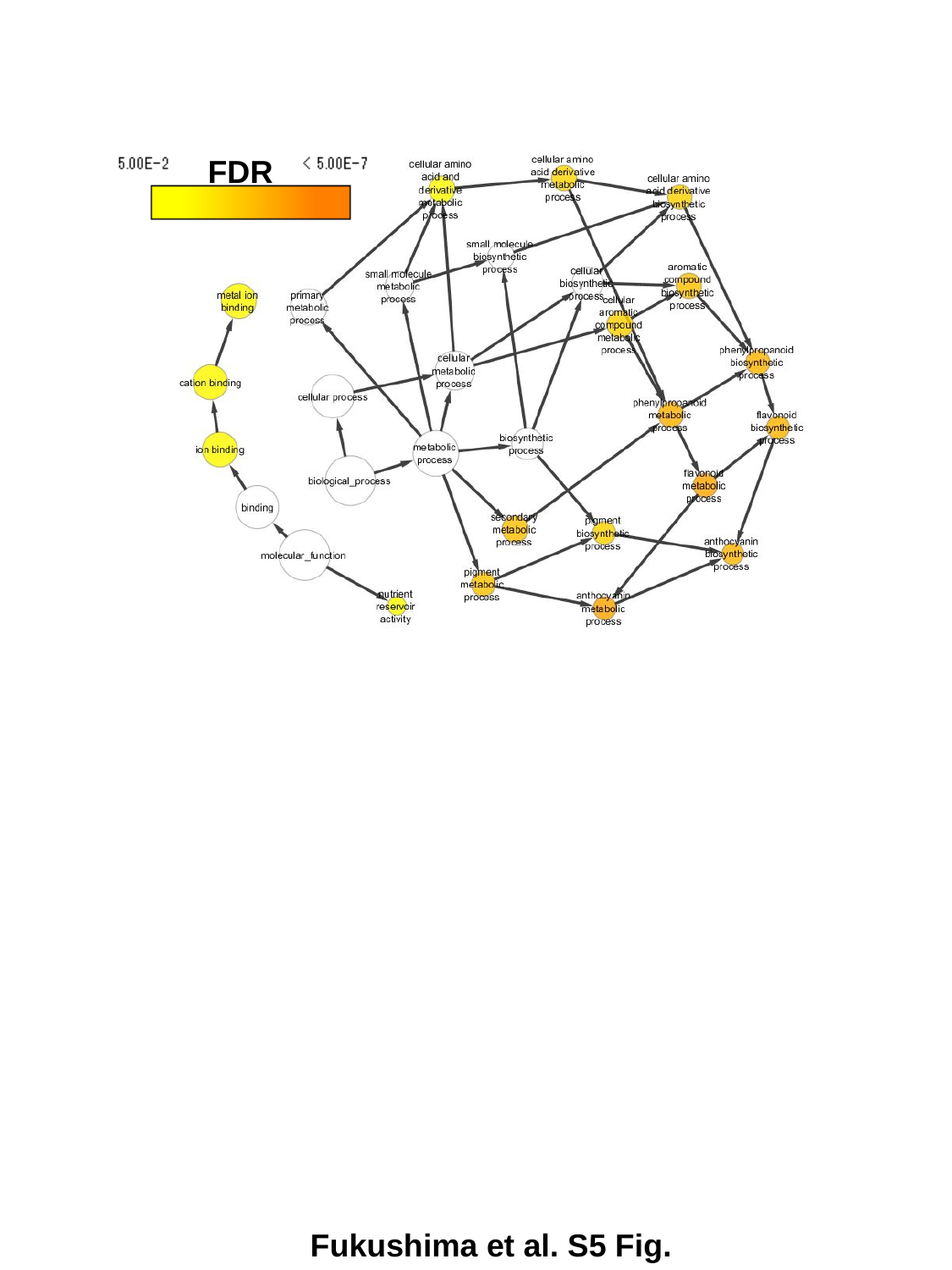

FDR
Fukushima et al. S5 Fig.

Supplement: S5 Fig — The colored nodes indicate the significantly over-represented GO categories. The pseudo-colored bar represents the significance [false discovery rate (FDR)]. (PPTX) [file pone.0129154.s005.pptx]
