# Supplementary material for: High-Throughput Sequencing and De Novo Assembly of Red and Green Forms of the Perilla frutescens var. crispa Transcriptome
Source: PLoS One. 2015 Jun 12;10(6):e0129154. doi: 10.1371/journal.pone.0129154 (PMC4466401; doi:10.1371/journal.pone.0129154)
Supplement: S3 Fig — Results summarized in three functional categories: cellular component, molecular function, and biological process. 29,813 unigenes were categorized by GO terms. The GO terms were visualized using WEGO (http://wego.genomics.org.cn) [39]. (PPTX) [file pone.0129154.s003.pptx]

## Slide 1
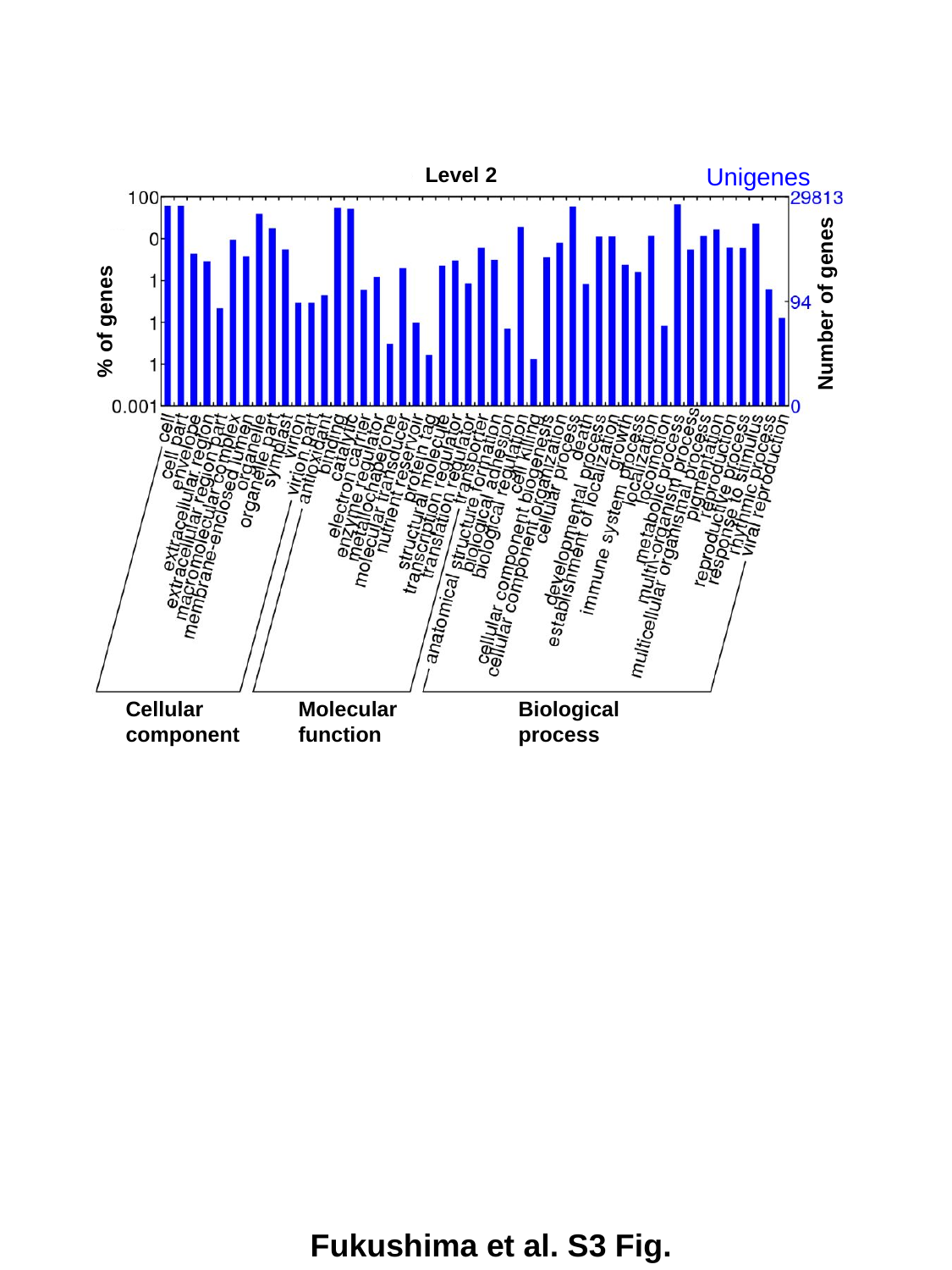

Unigenes
Number of genes
% of genes
Cellular component
Molecular function
Biological process
Level 2
Fukushima et al. S3 Fig.
