# Supplementary material for: High-Throughput Sequencing and De Novo Assembly of Red and Green Forms of the Perilla frutescens var. crispa Transcriptome
Source: PLoS One. 2015 Jun 12;10(6):e0129154. doi: 10.1371/journal.pone.0129154 (PMC4466401; doi:10.1371/journal.pone.0129154)
Supplement: S4 Fig — Annotated unigenes were grouped into 137 KEGG pathways. The top 30 pathways containing unigenes are displayed. (PPTX) [file pone.0129154.s004.pptx]

## Slide 1
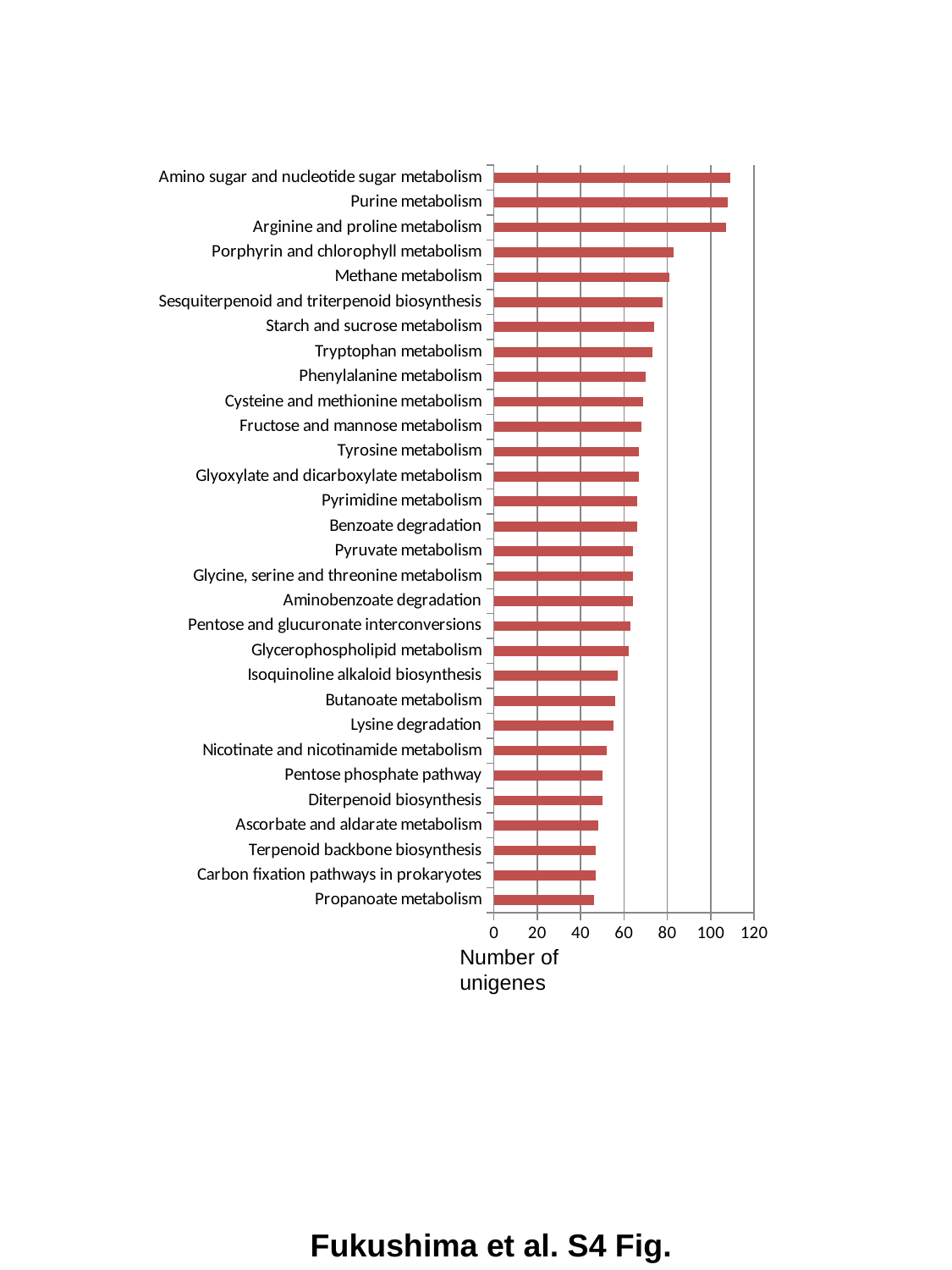

### Chart
| Category | |
|---|---|
| Propanoate metabolism | 46.0 |
| Carbon fixation pathways in prokaryotes | 47.0 |
| Terpenoid backbone biosynthesis | 47.0 |
| Ascorbate and aldarate metabolism | 48.0 |
| Diterpenoid biosynthesis | 50.0 |
| Pentose phosphate pathway | 50.0 |
| Nicotinate and nicotinamide metabolism | 52.0 |
| Lysine degradation | 55.0 |
| Butanoate metabolism | 56.0 |
| Isoquinoline alkaloid biosynthesis | 57.0 |
| Glycerophospholipid metabolism | 62.0 |
| Pentose and glucuronate interconversions | 63.0 |
| Aminobenzoate degradation | 64.0 |
| Glycine, serine and threonine metabolism | 64.0 |
| Pyruvate metabolism | 64.0 |
| Benzoate degradation | 66.0 |
| Pyrimidine metabolism | 66.0 |
| Glyoxylate and dicarboxylate metabolism | 67.0 |
| Tyrosine metabolism | 67.0 |
| Fructose and mannose metabolism | 68.0 |
| Cysteine and methionine metabolism | 69.0 |
| Phenylalanine metabolism | 70.0 |
| Tryptophan metabolism | 73.0 |
| Starch and sucrose metabolism | 74.0 |
| Sesquiterpenoid and triterpenoid biosynthesis | 78.0 |
| Methane metabolism | 81.0 |
| Porphyrin and chlorophyll metabolism | 83.0 |
| Arginine and proline metabolism | 107.0 |
| Purine metabolism | 108.0 |
| Amino sugar and nucleotide sugar metabolism | 109.0 |Number of unigenes
Fukushima et al. S4 Fig.
