# Supplementary material for: High-Throughput Sequencing and De Novo Assembly of Red and Green Forms of the Perilla frutescens var. crispa Transcriptome
Source: PLoS One. 2015 Jun 12;10(6):e0129154. doi: 10.1371/journal.pone.0129154 (PMC4466401; doi:10.1371/journal.pone.0129154)
Supplement: S6 Fig — Previously identified genes encoding transcription factor(s) and enzymes, and the expression of unigenes are shown. The expression levels (TMM-normalized FPKM values) are displayed. Homologous genes in red and green perilla indicate the reciprocal best-hit BLAST results. Asterisks represent the false discovery rate (FDR) < 0.05 obtained with the TCC package [43]. TMM, Trimmed Mean of M values [63]. (PPTX) [file pone.0129154.s006.pptx]

## Slide 1
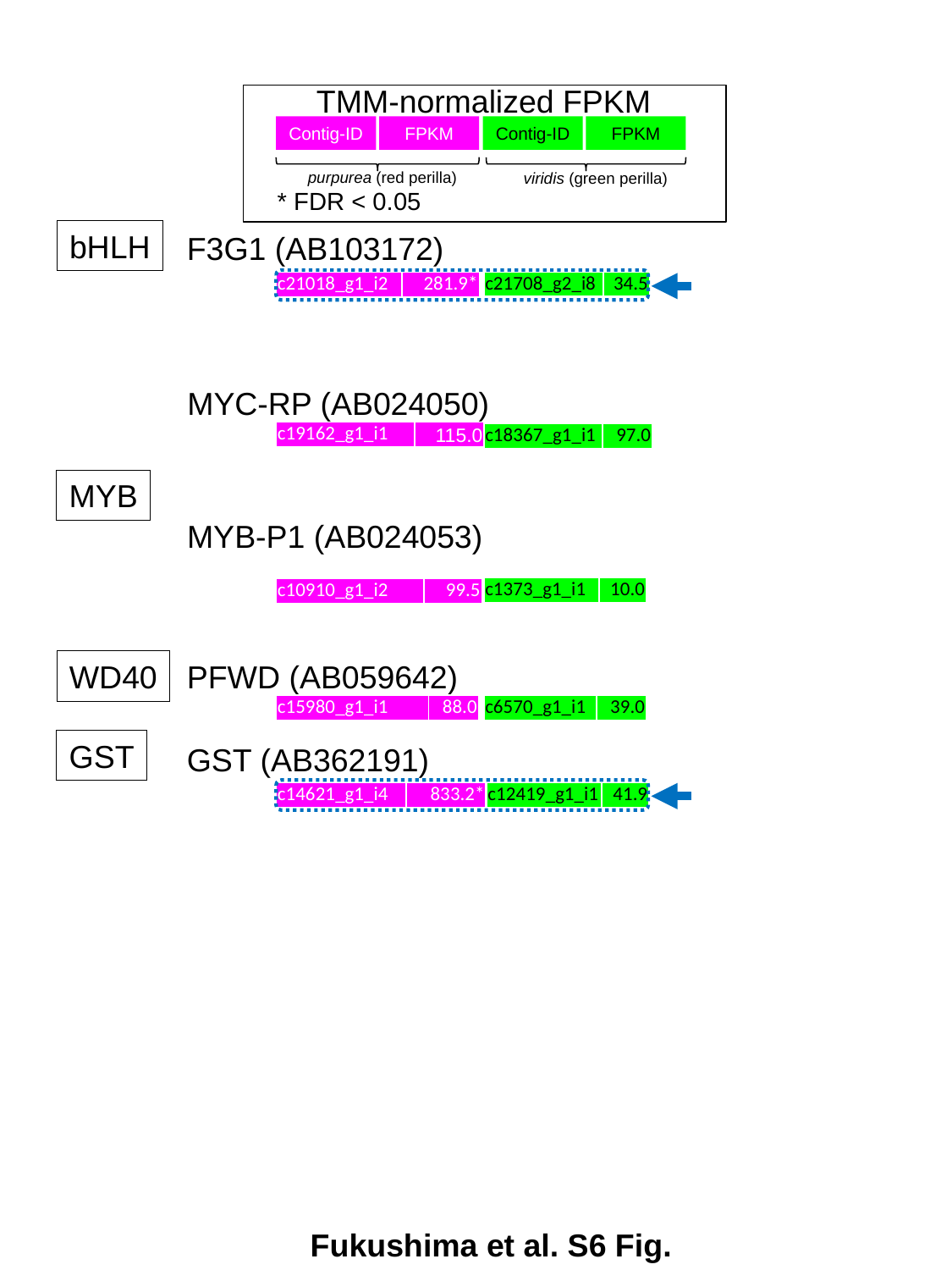

TMM-normalized FPKM
Contig-ID
FPKM
Contig-ID
FPKM
purpurea (red perilla)
viridis (green perilla)
* FDR < 0.05
bHLH
F3G1 (AB103172)
| c21708\_g2\_i8 | 34.5 |
| --- | --- |
| c21018\_g1\_i2 | 281.9\* |
| --- | --- |
MYC-RP (AB024050)
| c19162\_g1\_i1 | 115.0 |
| --- | --- |
| c18367\_g1\_i1 | 97.0 |
| --- | --- |
MYB
MYB-P1 (AB024053)
| c1373\_g1\_i1 | 10.0 |
| --- | --- |
| c10910\_g1\_i2 | 99.5 |
| --- | --- |
WD40
PFWD (AB059642)
| c15980\_g1\_i1 | 88.0 |
| --- | --- |
| c6570\_g1\_i1 | 39.0 |
| --- | --- |
GST
GST (AB362191)
| c14621\_g1\_i4 | 833.2\* |
| --- | --- |
| c12419\_g1\_i1 | 41.9 |
| --- | --- |
Fukushima et al. S6 Fig.
Fukushima et al. S6 Fig.
